# Managers and Hygiene Representatives' Perceptions of a Patient Safety Initiative to Reduce Healthcare‐Associated Infections: A Mixed‐Methods Study

**DOI:** 10.1002/hsr2.70572

**Published:** 2025-03-18

**Authors:** Eva Sving, Katarina Wijk, Maria Lindberg

**Affiliations:** ^1^ Centre for Research and Development Region Gävleborg/Uppsala University Gävle Sweden; ^2^ Department of Public Health and Caring Sciences Uppsala University Uppsala Sweden; ^3^ Faculty of Health and Occupational Studies, Department of Caring Science University of Gävle Gävle Sweden; ^4^ Faculty of Health and Occupational Studies, Department of Occupational Health Sciences and Psychology University of Gävle Gävle Sweden

**Keywords:** cross infection, delivery of health care, implementation science, patient safety, quality improvement

## Abstract

**Background and Aims:**

More knowledge about perceptions of implementing new ways of working to prevent organism transmission and create safety engagement in health care are needed. This study aimed to explore managers and hygiene representatives', in the role as facilitators, perceptions of safety engagement and factors of importance when implementing measures to reduce healthcare‐associated infections.

**Methods:**

Data were collected using both a quantitative and qualitative approach. A total of 24 facilitators were involved in the implementation process (12 managers, and 12 hygiene representatives, all female). The facilitators responded to the Sustainable Safety Engagement Index at three occasions, and 13 of the facilitators participated in open‐ended semi‐structured interviews.

**Results:**

The results displayed that both internal and external organizational factors affected the implementation process as well as the interactions between individuals within the organization. The Sustainable Safety Engagement Index did not indicate any deviations before and during the implementation process.

**Conclusion:**

To create a patient safety culture and get healthcare personnel engaged, it is important for healthcare managers to be aware of the complexity of healthcare and adapt organizational factors and specific elements in the caring chain. A systematic implementation approach, and reliable measurements along with use of single or multiple strategies is recommended. Furthermore, dedicated facilitators who creates an environment of support and cooperation between different professions and provides inspiration is crucial to maintain the improvement work. Prevailing behaviors should also be considered when planning and implementing patient safety interventions.

## 1 Introduction

Implementing new ways of working within healthcare is commonly described as challenging owing to the complexity of implementation processes in relation to the context [1]. To succeed with improvements in healthcare, it is important to use strategies to identify barriers and enabling factors within the organization before the implementation process begins [2, 3]. Moreover, a systematic approach to implementation that includes the participants' view is recommended to achieve success [3, 4]. The conceptual Promoting Action on Research Implementation in Health Services (PARIHS) framework was developed based on the idea that research results are rarely applied clinically, which is often due to barriers in the local setting [5]. The framework has evolved over time, and in the revised integrated (i‐PARIHS) framework, successful implementation is defined in relation to the attainment of project goals and results from facilitating innovations with the recipients in their specific context [6]. Research regarding implementation science presents alternative ways of handling difficulties when implementing something new in healthcare settings. The quality improvement framework (QIF), for instance, describes a synthesis of 14 critical steps and actions in an implementation process that can be used to identify barriers and enabling factors. The steps comprise four phases: (1) initial considerations regarding the host setting, (2) creating a structure for implementation, (3) ongoing structuring once implementation begins, and (4) improving future applications [7].

One area of specific importance to acknowledge is how to implement new ways of working to prevent organism transmission and create safety engagement in healthcare since healthcare‐associated infections (HCAI) and their prevention are judged to be a worldwide challenge. Globally, 7–10 out of every 100 hospitalized patients are affected each year. Common HCAIs are urinary tract infections, blood stream infections, and respiratory infections [8]. The burden of HCAIs leads to high overall human costs in terms of morbidity and mortality as well as high financial costs for the healthcare system itself [9]. It is estimated that 30%–70% of all HCAIs could be prevented [9, 10]. In Sweden, approximately 5% of hospitalized patients undergoing somatic care are affected by HCAIs annually [11]. Since this represents 57,000 patients, efforts is required to improve this situation to increase the patients' safety while undergoing care. This includes preventing errors and protecting patients from adverse events associated with healthcare [12]. To be successful in planning improvements, Nilsen et al. [3] describe the importance of gaining insight into the healthcare personnel's (HCP) views to minimize barriers and maximize what facilitates behavioral change. Furthermore, it is important to acquire knowledge regarding HCP's perceptions of how they evaluate patient safety in terms of other competing priorities at work [13].

DiCuccio [14] presents several relevant factors to consider when establishing behavioral norms at work, which is also called the patient safety culture. These factors include shared values among colleagues, their beliefs concerning how to manage the organization, and how these values and beliefs interact with the units and the organizations' structures and systems. A positive patient safety culture has been described as associated with higher hand hygiene compliance [15], which has been considered the most cost‐effective way to reduce HCAIs [16, 17]. However, compliance with hand hygiene measures is challenging in healthcare settings [18], and there is a need for shared responsibility regarding the quality of care among HCP and managers [19]. Owing to the complexity of healthcare and how to successfully improve the quality of patient care, it is important to evaluate planned implementation processes and to continuously learn from them. Thus, this study was designed to explore managers and hygiene representatives' safety engagement and their perceptions of an HCAIs implementation process to reduce HCAI, when acting as facilitators.

## 2 Material and Methods

The present study used a prospective design [20]. The Consolidated Standards of Reporting Trials was used as a checklist [21] and is available in Supporting Information S1: Additional file 1.

### 2.1 Setting

The operational area that implemented measures to reduce HCAIs involved 12 specialized medical care units consisting of 5 wards and 7 outpatient clinics, 2 units for physicians, and 1 administrative unit. In total, the operational area employed approximately 450 individuals from different professional backgrounds, such as physicians, registered nurses, licensed practical nurses, and administrative personnel. Each care unit was led by a first‐line manager (FLM) who, depending on the number of employees, had an assistant FLM. At least one employee was designated the role as a hygiene representative at respectively care unit. The hygiene representatives' responsibilities are described in Table 1.

**Table 1:** Overview of the topics from the five joint meetings to increase the facilitators’ knowledge of the improvement work process, determine the involvement of all employees, and definition of responsibilities for hygiene representatives.

**Phase and focus of implementation**	**Time period**	**Subprocess in the improvement work**	**The meeting topics for training and discussion**	**Participants**
Initial considerations: What is being implemented	Jan 2019	Define “What to do” and setting goals, defining the role of hygiene representative*	Safe care for patients	Facilitators, Head of the operational area
Creating a structure: How will it be implemented	Feb–May 2019	Define “How to do it”	Involvement (One full‐day for each employee in groups)	All employees in the operational area
Cont. creating a structure: Who will be targeted, Where will it be implemented	April 2019	Identify and plan for measures and improvements	Workflow	Facilitators, Head of the operational area
Ongoing structure: To organize improvement work	Aug–Sep 2019	Identify and plan for measures and improvements	Methodology for improvement work	Facilitators, Head of the operational area
Ongoing structure: To organize improvement work	Feb 2020	Conduct improvement work	Change management	Facilitators, Head of the operational area
Improving future applications: To maintain and improve results	Oct 2020	To complete improvement work	Sustainability	Facilitators, Head of the operational area

* The responsibilities of a hygiene representative were described as: Responsible for carrying out measurements of basic hygiene routines and dress code (BHK) and healthcare‐associated infections (VRI). Report back the results of BHK and VRI measurements and inform about current events and guidelines, for example, at a hygiene station, regularly scheduled workplace meeting or morning meeting. Play an active role in the induction program by informing new staff about healthcare hygiene procedures. Participate in educational hygiene rounds and provide feedback to all staff. Provide support on hygiene issues for all staff groups, including external groups, for example, paramedics. Be a role model for others in their work. Encourage discussion by providing constructive feedback to staff. Participate in the forum for hygiene representatives in internal medicine. Participate in hygiene‐representative meetings organized by the local unit for Infectious Disease Control.

**Figure 1:**
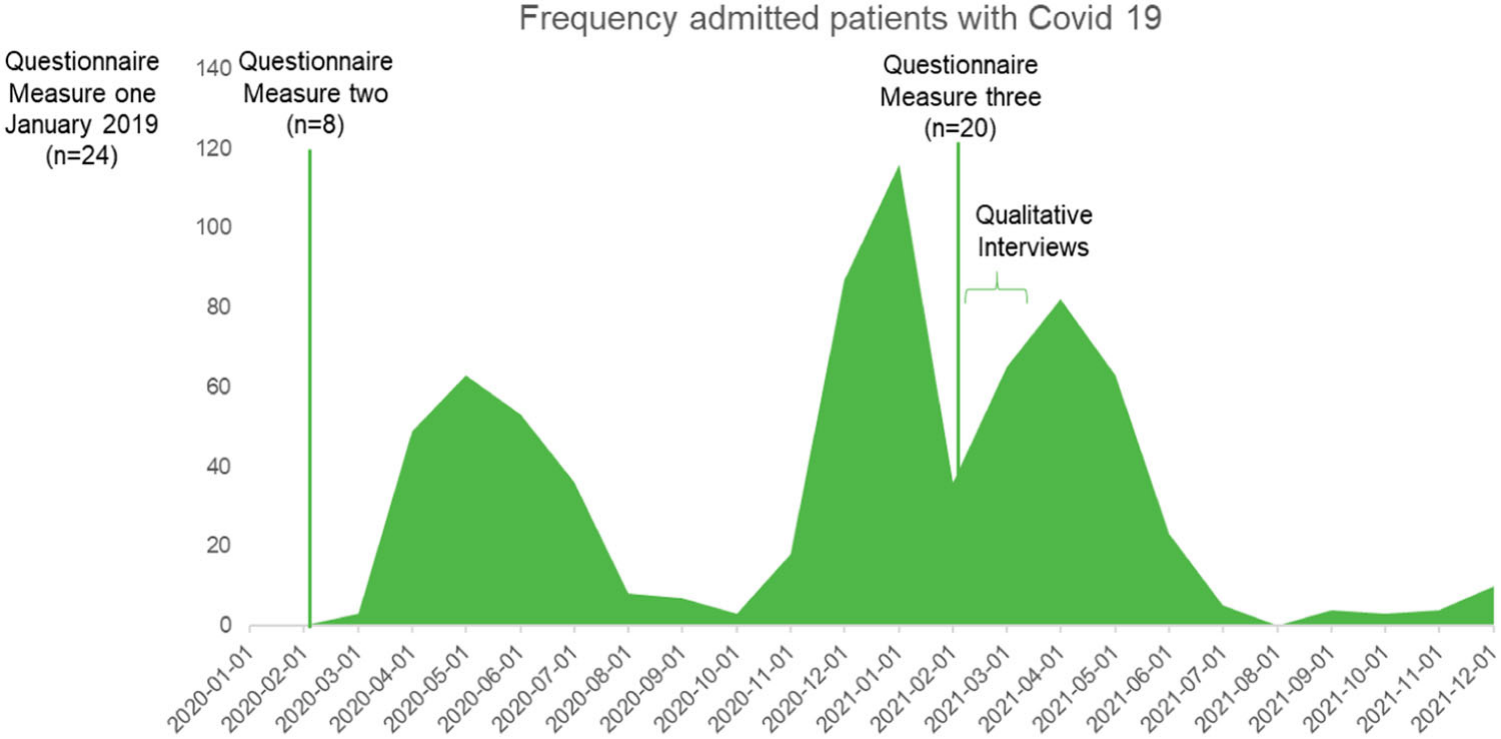
Overview of the frequency of admitted patients with Covid‐19 on the first day of each month during the years 2020–2021 in the hospital together with time points for data collection.

The QIF was used as a guide for organizing the implementation process [4], and a total of 24 FLMs and hygiene representatives were assigned the role as facilitators. To increase the facilitators' knowledge of managing improvement work and implementing the required improvements, five meetings were planned and spread out during the prearranged time for conducting the improvement work. For information on the meetings' content, see Table 1. In the start of the implementation process, all the HCP within the operational area were invited to participate in one all‐day meeting to discuss the preconditions for safe care and their involvement in the improvement work. While the implementation process was in progress, the Covid‐19 pandemic developed, and the hospital occupancy varied from around 10 patients to around 120 patients with a Covid‐19 diagnosis during 3 intensive periods (Figure 1). During the Covid‐19 pandemic it was necessary to establish many priorities at the hospital level. For instance, specific units were opened to care for patients having symptoms of Covid‐19, signifying that ordinary patient beds were no longer available, and personnel (from all occupational categories) were assigned to these specific beds.

### 2.2 Participants

All 24 facilitators consisting of 12 FLMs and 12 hygiene representatives, all female, were invited to participate in the study survey at the start of the improvement work process. Of these, 13 also agreed to participate in the qualitative interviews (seven FLMs and six hygiene representatives) during the implementation work process.

### 2.3 Data Collection

Data were collected from the facilitators using both a quantitative and a qualitative approach. The Sustainability Safety Engagement questionnaire [22] consisting of eleven items assessed on a scale of 1–5, where option 5 was “completely agree,” was used to quantitatively collect data. Face and content validity for the items in the questionnaire was judged by experts and researchers in the field during the process of developing the questionnaire, produced by The Swedish Association of Local Authorities and Regions. The questionnaire was further tested for face validity by healthcare personnel and is commonly used by regions in Sweden to follow up on patient safety. The nine items included in the Sustainable Safety Engagement index were used in this study:

1) Second‐line managers provide the first‐line managers with the conditions for conducting safe care.
2) At my workplace, we learn from what works well.
3) At my workplace, we always act based on the risks we see.
4) At my workplace, improvements are always made after negative events (a negative event entail something undesirable).
5) I alert others when I think something is about to go wrong.
6) I dare to talk about my mistakes.
7) I am always treated well at my workplace when I need help.
8) At my workplace, we cooperate well with other units.
9) At my workplace, we adapt the work so that safety is preserved when conditions change.

The questionnaire was handed out three times, two times before the pandemic occurred and once during the pandemic (Figure 1).

Qualitative data were collected through open‐ended semi‐structured interviews in February–March 2021 (Figure 1). The presumptive informants were informed about the study procedure in January, and informed consent was obtained from those who agreed to participate before the interview were conducted. The FLMs were interviewed individually by one of the researchers (ES). The hygiene representatives were interviewed in couples, and two of the authors (ES, who moderated the interview, and ML, who acted as an assistant moderator) conducted these interviews. Both authors had experiences in the field and in performing qualitative interviews. An interview guide was used in all interviews to ensure that all participants were asked about the respective content areas. The interview guide was developed based on the i‐PARIHS framework and its four core constructs: facilitation, innovation, recipients, and context 6]. Clarifying questions such as “Please, explore that” were used when further information was needed to understand the respondents' descriptions. The interviews were conducted digitally; audio recorded and lasted between 32 and 54 min, with an average time of approximately 46 min. Malterud et al. [23] suggest that a sample with high information relevance requires fewer participants to achieve sufficient information power. Our study, with a narrow aim, used purposive sampling, involving all eligible facilitators, 12 first‐line managers, and 12 hygiene representatives engaged in the quality improvement work. Given the aim, specific questions, and dialog, we estimated that interviews with ten facilitators would suffice but conducted interviews with 13 individuals as the opportunity allowed.

### 2.4 Analysis

The self‐ratings of the nine items in the questionnaire are summarized in an index created by taking the mean of all items. The 1–5 scale was transformed into a 1–100 scale when calculating the index. The index (mean) of the facilitators answers was presented descriptively using SPSS 25.0. A descriptive figure was conducted, illustrating the ratings for each item. This to follow the implementation process. Since the group of facilitators was small, it was decided that the results should only be presented with descriptive statistics, therefore no reporting on significance was made.

The inductive qualitative content analysis was begun during the data collection phase and conducted in Swedish in accordance with the description by Graneheim and Lundman [24] by the first and last authors. The transcribed interviews were read several times to gain an understanding of the content. The key findings were highlighted, condensed, and assigned a code to capture the essential meaning of the text by using a word or a sentence. The codes were compared for similarities and differences, and codes that were interpreted as belonging together were divided into sub‐categories. These sub‐categories were then classified into categories. To identify the underlying content of the text, all the transcripts were reread and interpreted in a theme. Excerpts were identified for illustration of the findings. We used tables in Word to sort the data during the analysis process. The analysis was performed as a dynamic process and continuously discussed by all the authors.

### 2.5 Ethics Approval and Consent to Participate

The Swedish ethical review authority approved the study protocol (Reg. no. 2019/04031). A written information about the study was sent by email to the participants and written informed consent was obtained from all participants who agreed to participate. Two of the researchers had had a professional relationship with some of the participants. The listed authors are those entitled to authorship according to ICMJE uniform requirements for manuscripts.

## 3 Results

The facilitators' safety engagement and perception of the HCAI implementation process was interpreted in the theme “Requirement of trustful collaboration and contextual knowledge to manage successful implementation.” The three categories and six sub‐categories that support the theme along with the excerpts are presented in Table 2. The results from the Sustainable Safety Engagement Index are presented separately below.

**Table 2:** Overview of the excerpts, subcategories, categories, and interpreted theme from the interviews with the facilitators.

Excerpts	Subcategory	Category	Theme
*… who was before me and here who (.) was a hygiene representative, everyone pretty much knew that she was a hygiene representative and (.) and when she came then [measuring compliance] everyone shaped up. (.) And (.) then we said, yeah, but it might be good to (.) have someone they don't know‐ (.) (hygiene representative)*	Measurements to improve care	Understanding the need for change	Requirement of trustful collaboration and contextual knowledge to manage successful implementation
*[Activity day] It should be like you feel somehow (.) involved in‐, in this we've also been involved in deciding, so it's not just us sitting around deciding something…//… They would become involved (.) and perhaps think that it was uh, was (.) more important then (.) perhaps…//… And we have also tried to do so at the workplace meetings. (hygiene representative)*	A systematic approach to achieve change		
*it's always a‐ a trade‐off and uh (.) what‐ what should I prioritize. It is ‐ is (.) digital care meetings, is it (.) salary reviews and (.) and things like (.) [00:12:15] Unfortunately, it can be the case that (.) a project like this (.) may be prioritized less even though it should actually be prioritized more. Because you ‐ you ‐ see the time crunch then. (.) Everything‐ everything‐ everything is important. (First‐line manager)*	Planning for unit‐based work	Dealing with contextual conditions	
*Because (.) most people have‐ they‐, (.) everyone knows (.) there's no one who doesn't know (.) about healthcare hygiene. (.) They‐ that's not what this is about. (.) Instead, (…) it's different ‐ it's about not doing the right thing. (…) (First‐line manager)*	Awareness of behaviors to develop safe care		
*it is very important that the manager is involved in this. Because (.) I think (.) the staff will be more motivated (.) to (…) follow the advice or yes, (.) do their absolute best. (.) It comes from the top. (hygiene representative)*	Motivation is a prerequisite for change	Changing together	
*I've got my eye on it, but that they see. I mean, they are the ones who do the m‐, the BHK measurements. They are the ones who see what we need to improve. (.) And then, that I provide support and (.) also make suggestions about (.) w‐ w‐ where we could work with. (First‐line manager)*	Collaboration between roles for success		

### 3.1 Understanding the Need for Change

This category consists of two subcategories that denote the importance of having reliable measures for performing improvement work and the usefulness of having a systematic approach for planning, carrying out, and evaluating the improvement activities.

#### 3.1.1 Measurements to Improve Care

The respondents stated that indicators to measure quality improvements were important, demanding, and an objective way to evaluate needed improvements. However, they indicated that it was difficult to interpret the results and understand how the 2 monthly assessed indicators, that is, compliance to hand hygiene measures, including protective clothing (referred to as compliance below), and the level of HCAIs, were associated. The respondents also described it difficult to find new appropriate measures that were interesting and not considered dull for the activities. Chosen indicators were, for example, repeated sample collections from visitor screens and walking aids, and short questionnaires to patients.

Compliance was initially monitored openly but was then “blinded,” signifying that the observed person did not know that they were being observed; this led to strikingly lower compliance rates. It was considered difficult to reach 100% compliance owing to that there was always someone missing a part because there are many people and steps involved. The respondents perceived, however, that HCP compliance increased when the risk of organism transmission to themselves was judged to be high, for example, before they entered the personnel room. Measuring compliance was regarded as time consuming and problematic at the outpatient clinics since HCP meet the patients one‐on‐one.

HCAIs were perceived as an indicator showing how safe the care was for the patients. The respondents described it as challenging to assess the level of HCAIs since many patients' contract HCAIs due to immunosuppressive treatment. The respondents indicated that they had access to a national quality register for HCAIs, but they did not know how to use it and regarded it as unreliable. The pandemic was perceived to have affected the level of HCAIs positively since HCP became more careful with conducting hygiene measures.

#### 3.1.2 A Systematic Approach to Achieve Change

The respondents stated it was crucial to carry out the improvement work using a systematic work process that included feedback on the results, joint decisions about required improvement activities, and following up on the effects. If the intended effects were not attained, new activities were selected. Oral feedback on the indicator results was provided during workplace or morning meetings, and written information was either sent via e‐mail to all HCP or posted on a designated bulletin board at the unit. The physicians only got feedback on the results on a few occasions since the FLMs at the care units were not their managers. Results from the indicators were described as used for planning additional improvement activities. Activities described as preventing organism transmission were applied to the HCP's workflow for the caring rooms, material handling, and caring processes. The pandemic had, however, made it less possible to work systematically.

#### 3.1.3 Dealing With Contextual Conditions

This category includes descriptions of the need for contextualization when planning improvement activities, and the necessity to consider behaviors among the HCP in the units' improvement work.

#### 3.1.4 Planning for Unit‐Based Work

When planning for activities, the respondents perceived it important to consider the units prerequisites; therefore, different activities were described as required in each respective unit. The hygiene representatives perceived they had a significant influence on the improvement work in their unit as they provided suggestions for activities at workplace meetings, which were accepted by the HCP.

The respondents described it as difficult to get the time they needed for planning. When the hygiene representatives had to prioritize between the improvement work and work with patients, they always prioritized the patients. Consequently, they planned the improvement work while performing their ordinary daily work. The FLM also had to prioritize different work tasks, which often resulted in not prioritizing the improvement work. However, they perceived that it was easier to prioritize the improvement work since the head of the operational area was the initiator.

The joint meetings with all facilitators were described as essential even if it was difficult to take time out for them. The respondents described the need to be prepared for the meetings since a recurring point on the agenda was to present a report on the activities conducted since the last meeting and the plan forward. The meetings created energy to continue the work, and any new ideas discussed were brought back to the units. However, presenting the new ideas to the HCP was considered troublesome since they had not participated in the discussion.

#### 3.1.5 Awareness of Behaviors to Develop Safe Care

Factors perceived as affecting the risk of organism transmission were the environment, the patients, and the HCP's understanding of work processes designed to keep themselves, materials, and the environment clean. Good teamwork, to be helpful, and to be able to express oneself was regarded to benefit creativity to develop safe care. Individual advice to HCP on how to improve compliance to different work processes was described as contributing to enhanced patient safety. However, some HCP reacted adversely, and pedagogical skills were requested. To work with the patient to prevent risks for organism transmission through individual care plans, was described important to provide clear information and listen to the patient. Knowledge from new colleagues were seen as important since they could influence others by providing new perspectives. It was, however, regarded as necessary to provide HCP with continuous training in safe practices.

High turnover, a lack of work experience, and limited knowledge were attributed to decreasing patient safety. Stressful situations were perceived as generating low compliance with safe work procedures, such as hand disinfection before patient contact. The respondents indicated that HCP questioned the evidence regarding when to use gloves in care situations. To cooperate with an external unit in improvements was deemed difficult owing to that no one had the authority to decide on the necessary investments.

### 3.2 Changing Together

Implementing change activities together was perceived as motivating and an important prerequisite for moving forward. To feel support and cooperation from others with different roles provided further inspiration to continue the improvement work.

#### 3.2.1 Motivation Is a Prerequisite for Change

Several conditions were regarded as important in strengthening the improvement work: the head of the operational area initiated it and articulated a clear goal in an important area, the FLMs commitment, familiar indicators for feedback of results, the five meetings for facilitators, and the full day to involve all HCP in the improvement work. Having joint discussions about improvement activities with the HCP made them more engaged and aware about how to provide safer care. They also became more open to test ideas, and fulfillment increased. Moreover, when different HCP performed the compliance audits of hygiene measures, awareness of its relevance increased among the HCP.

The respondents believed that better communication regarding why the improvement work was prioritized could have further increased the HCP's understanding and motivation. The physicians were described as motivated by evidence, and the use of other indicators could have increased their engagement. To boost the HCP's motivation, different activities were considered important, such as a “hygiene week” twice a year with specific events. Moreover, the respondents stated that no investment in cleanable furniture was allowed by top management, which created a lack of motivation among the HCP. Different managers for the physicians, registered nurses, and licensed practical nurses were perceived to make it difficult to get everyone involved in the improvement work. Subsequently, the physicians didn't participate in the same meetings as the nursing personnel, and the respondents considered they had insufficient knowledge regarding the physicians' reasoning about the improvement work.

#### 3.2.2 Collaboration Between Roles for Success

The FLMs' role was described as important for the improvement work since they had knowledge and experience in the field, in setting goals, and knowledge about routines. FLMs organized and directed the work and provided the needed prerequisites and support. The respondents stated that an engaged FLM influenced the HCP with their positive attitude, and they made it possible for the hygiene representatives to take part in the arranged meetings with all the facilitators. Those meetings provided time for reflection, and increased cooperation between the units. Listening to others provided inspiration, and describing their own unit's work at the meetings made them proud.

The role of the hygiene representatives was also described as important in the improvement work. Work assignments were given by the FLM, and if problems arose they solved it together. The FLMs expressed that the hygiene representatives took on much responsibility, conducted activities, showed good knowledge, and provided support to the HCP. However, they expected more initiative from the hygiene representatives in terms of scheduling time to work on the quality improvements and notifying the FLM of the results. If a FLM or hygiene representative finished their respective work assignment, the one remaining felt that their responsibility for the improvement work increased. Being a hygiene representative was considered difficult in times of high turnover since they were involved in teaching new employees how to perform and comply with the various routines. They would have liked more support from the local unit for Infectious Disease Control in terms of how to best prevent organism transmission.

### 3.3 Sustainable Safety Engagement Index

**Figure 2:**
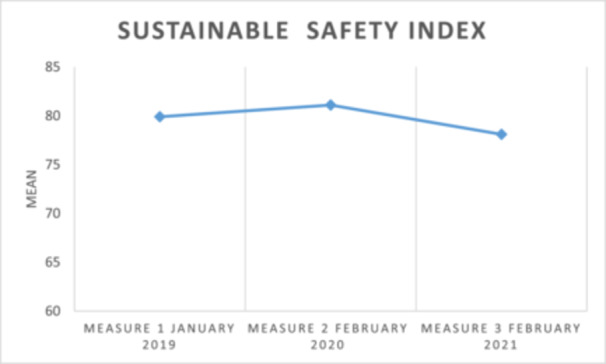
The Sustainable Safety Engagement Index at baseline, and 1 and 2 years after the improvement work started.

The index of sustainability Safety Engagement, measured among the facilitators before and during the improvement work showed no substantial variation, as shown in Figure 2. The index ranges between 81.1 and 78.1, which is considered high [22].

**Figure 3:**
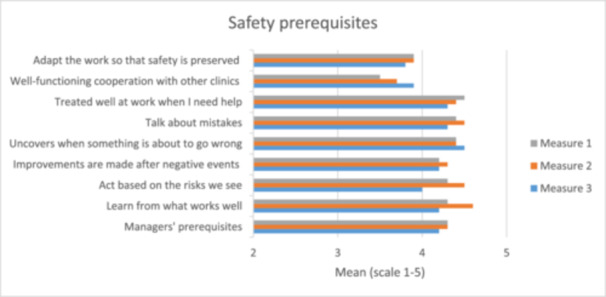
Results from the questions on Sustainable Safety Engagement among the facilitators.

No major difference between the measurements was seen regarding the issues of safety engagement, and the collaborative experience with other clinicians seemed to have increased. However, a downward trend in sustainability engagement was seen in six of the nine items from the first to the third measurement (Figure 3).

## 4 Discussion

The results from the interviews indicated that both internal and external organizational factors affected the implementation process as well as the interactions between individuals within the organization. To understand the need for change and relate to the contextual conditions of one's own unit and still carry out the change together was described as beneficial for successful implementation. However, making this happen was considered challenging. The Sustainable Safety Engagement Index did not indicate deviation from before and during the implementation process.

The operational area used the QIF [4] as a guide to plan and implement the improvement work. Activities performed were described as having a positive impact on the implementation process. Thus, including a framework such as the QIF [4] in the planning process seems effective. McElearney et al. [25] describe three aspects for successful implementation in an organization: (1) engagement of executive leadership, (2) information sharing, and (3) manager coaching, which is in line with the planned implementation process in the current study. Another framework that provides potential for successful implementation is the i‐PARIHS framework, which emphasizes facilitation as the main construct during the implementation process [6]. Facilitation is described as important to overcome obstacles in implementation work, and as a facilitator, it is beneficial to have the personal characteristics and skills to master the tasks [26]. Therefore, a facilitator should be carefully selected based on the specific obstacles identified before implementation. In this study, FLMs and hygiene representatives were assigned to be facilitators. Neither the FLMs nor the hygiene representatives had enough dedicated time for facilitating the improvement work and thus the implementation process. Implementation research states that resources must be secured before the start of an implementation project [4, 27], in this case in the form of time. Another factor that had a negative impact on allocating time during the implementation process was the Covid‐19 pandemic. If resources like time are not considered during an implementation process, it can lead to both a lack of progress [26] and decreased work satisfaction.

To prevent HCAIs, it is important to assign accountability to both senior leaders and managers [28]. Gilmartin and Hessel [29] describe several frequently applied implementation strategies to limit HCAIs; audits and feedback, facilitation, and strategies tailored to address the hindering and enabling factors identified in previous data collection [29]. Such multiple strategies were included in the current improvement work, and the facilitators had the responsibility to accomplish them. Thus, when conducting improvement work, the use of single or multiple strategies should be considered. The i‐PARIHS framework states that the context determines which strategies are needed [6]. Even though the facilitators said that the strategies used had a positive impact on the implementation process, they also pointed out obstacles in the organization. During the implementation process, these obstacles were not analyzed or evaluated to determine if there was a need to test other strategies to move the implementation process forward. Considering the obstacles identified within the context is thus essential [4, 26, 29, 30] to reach success.

To prevent organism transmission and limit the number of patients that contract HCAIs, there was a need to change the way the HCP worked. The study results showed that to audit and give feedback on indicators motivated the HCP to change their behavior. Following indicators and providing feedback are described as important factors in successful implementation [4, 31, 32]. Notification of the monthly indicator measurements, be it orally, electronically, via bulletin board or person to person, is important to successfully change behavior [25]. Even though indicators were described as important, they also created some obstacles. For instance, the physicians had no trust in the evidence regarding compliance with hand hygiene measures, including protective clothing. Trusting evidence is one of three important factors for successful implementation [6]. The respondents stressed the importance of knowing how to perform a work task leading to a low risk of organism transmission. Being aware of the actual work processes involved in healthcare is important since written guidelines cannot be comprehensive and commonly fail to account for the dynamic nature of the work [33]. So, HCP must go through different work processes to evaluate the risk for organism transmission [34] before they start the work task, which can make them aware of the changes needed for improvement. This can also lead to the discovery of new specific indicators important for measuring safe care, but time and employee engagement are required.

As shown in this study, making changes to healthcare practices is complex, as is the case with implementation frameworks [4, 6]. Also, during this study, the Covid‐19 pandemic struck and most likely had some impact on the implementation process. The systematic nature of the work performed using an implementation plan made it possible to continue working even during the pandemic, and the facilitation from the FLMs and hygiene representatives was described crucial. The need for an external facilitator was, however, recognized since they could act as a link between the micro‐, meso‐ and macro levels, which is in correspondence with previous research [6, 12]. Patient safety signifies protecting patients from adverse events like HCAIs during the patients journey through the healthcare system. Teamwork as well as organizational and behavioral learning are described having an important impact on the patient safety culture [35]. Throughout this implementation work, only small change was seen in the Sustainable Safety Engagement Index. Due to the sample size available, this study cannot show changes in conditions for safe care. Nevertheless, the implementation of evidenced‐based care is key; patients should not be harmed, and every patient should always receive safe and respectful care everywhere [36].

### 4.1 Methodological Limitations

The second time the questionnaire was handed out few facilitators responded which make it difficult to draw conclusions from the survey. The third distribution of the questionnaire was initially planned to take place after the completion of the quality improvement work. However, due to the pandemic affecting the organization's ability to adhere to the original schedule, the final distribution occurred during the ongoing improvement process. The used questionnaire may also not have captured small differences concerning the facilitators Sustainable Safety Engagement. In the study's qualitative research approach trustworthiness was reflected on via credibility, dependability, and transferability [22]. To strengthen credibility the study aim guided the data collection, and data analysis. Just over half of the study participants took part in the interviews; conducted either as individual interviews or in pairs. It is possible that a different result would have been obtained if all facilitators had participated in both the study survey and the interview. Including participants who accurately represented the studied phenomenon, that is, facilitating an improvement work process, improved credibility. The hygiene representatives were interviewed in pairs to enable a deeper discussion about their safety engagement and their perceptions of the implementation process. The choice of using different approaches in the interviews with FLM (individual) and hygiene representatives (in pairs) may have affected the results. The interviews could be considered short, however, the informants provided rich descriptions to the study. All authors were involved in the analysis and the sub‐categories, categories and theme were discussed until a consensus was reached, enhancing dependability. Via our methodological description readers can evaluate the transferability of the findings.

## 5 Conclusions

To create a patient safety culture and get healthcare personnel engaged, it is important for healthcare managers to be aware of the complexity of healthcare and adapt organizational factors and specific elements in the caring chain. A systematic implementation approach, and reliable measurements along with use of single or multiple strategies is recommended. Furthermore, dedicated facilitators who creates an environment of support and cooperation between different professions and provides inspiration is crucial to maintain the improvement work. Prevailing behaviors should also be considered when planning and implementing patient safety interventions.

## Author Contributions

**Eva Sving:** conceptualization, methodology, validation, investigation, data curation, writing – original draft, writing – review and editing; project administration, resources, formal analysis, visualization. **Katarina Wijk:** conceptualization, methodology, validation, investigation, writing – original draft, writing – review and editing, resources, visualization, formal analysis. **Maria Lindberg:** conceptualization, methodology, validation, investigation, data curation, writing – original draft, writing – review and editing, resources, formal analysis, visualization.

## Conflicts of Interest

The authors declare no conflicts of interest.

## Transparency Statement

The lead author Eva Sving affirms that this manuscript is an honest, accurate, and transparent account of the study being reported; that no important aspects of the study have been omitted; and that any discrepancies from the study as planned (and, if relevant, registered) have been explained.

## Supporting information

Supporting information.

## Data Availability

The datasets generated and/or analyzed for the current study are not publicly available due to privacy or ethical considerations, but they are available from the corresponding author upon reasonable request.
